# Coexisting Irritable Bowel Syndrome and Overactive Bladder Are Associated with Increased Symptom Severity in Women with Fibromyalgia

**DOI:** 10.3390/healthcare14162515

**Published:** 2026-08-12

**Authors:** Ekrem Aslan, Duygu Kurtulus, Ferhat Yakup Suceken

**Affiliations:** 1 Department of Gastroenterology, Memorial Atasehir Hospital, Istanbul 34750, Türkiye; 2 Department of Physical Medicine and Rehabilitation, Faculty of Medicine, Memorial Atasehir Hospital, T.C. Üsküdar University, Istanbul 34662, Türkiye; dygkurtulus@gmail.com; 3 Department of Urology, Memorial Goztepe Hospital, Istanbul 34634, Türkiye; ykpsckn@gmail.com

**Keywords:** fibromyalgia, irritable bowel syndrome, overactive bladder, visceral hypersensitivity, central sensitization, symptom clustering

## Abstract

**Highlights:**

**What are the main findings?**

- Irritable bowel syndrome and overactive bladder symptoms commonly overlap in women with fibromyalgia.
- Patients with concomitant bowel and bladder symptoms exhibited greater overall fibromyalgia symptom burden.

**What are the implications of the main findings?**

- Bowel–bladder symptom clustering may characterize a subgroup of fibromyalgia patients with greater overall symptom burden.
- Recognition of coexisting gastrointestinal and urinary symptoms may support a more integrated clinical assessment in fibromyalgia.

**Abstract:**

**Background/Objectives**: Fibromyalgia (FM) is increasingly recognized as a multisystem disorder characterized by central sensitization and visceral symptom involvement. Irritable bowel syndrome (IBS) and overactive bladder (OAB), which share overlapping pathophysiological mechanisms related to altered visceral processing, are both commonly reported in FM. However, whether gastrointestinal and urinary symptoms cluster within the same patients and whether such clustering is associated with greater FM severity remain insufficiently explored. This study aimed to evaluate the overlap between IBS and OAB in women with FM and to determine whether their coexistence is associated with differences in FM severity. **Methods**: This cross-sectional observational study included 74 women with FM evaluated at the Physical Medicine and Rehabilitation outpatient clinic of Memorial Ataşehir Hospital between August and December 2025. FM severity was assessed using the Widespread Pain Index (WPI), Symptom Severity Scale (SSS), and FM Severity Score (GSS). OAB symptoms were evaluated using the Overactive Bladder Symptom Score (OABSS), and IBS status was determined based on prior clinical diagnosis. For subgroup severity analyses, patients were categorized as IBS + OAB, OAB only, or neither condition; the two patients with IBS alone were excluded from these comparisons because of the small cell size *(n* = 72). **Results**: IBS was present in 13 patients (17.6%) and OAB in 46 patients (62.2%). A substantial overlap between bowel and bladder symptoms was observed: 84.6% of IBS-positive patients had OAB, and IBS prevalence was higher in OAB-positive than OAB-negative patients (23.9% vs. 7.1%; OR = 4.56, 95% CI 0.93–22.24), although this association did not reach statistical significance (*p* = 0.063). Significant overall group differences were observed for SSS (*p* < 0.001) and GSS (*p* = 0.001), whereas WPI did not differ significantly across groups (*p* = 0.228). Patients with concomitant IBS and OAB had significantly higher GSS scores than both the OAB-only (adjusted *p* = 0.003) and neither-condition groups (adjusted *p* = 0.001). In multivariable analysis adjusted for age, concomitant IBS and OAB remained significantly associated with higher GSS (B = 3.77, 95% CI 1.59–5.95; *p* = 0.001). The regression model explained 18.2% of the variance in GSS (R^2^ = 0.182; *p* = 0.003). **Conclusions**: Concomitant IBS and OAB in women with FM were associated with greater overall FM symptom burden, particularly higher SSS and GSS scores, whereas widespread pain severity did not differ significantly across groups. The presence of either IBS or OAB may warrant evaluation for the other condition, as bowel–bladder symptom clustering may characterize a subgroup with greater overall symptom burden and support consideration of a more integrated clinical assessment.

## 1. Introduction

Fibromyalgia (FM) is a chronic pain disorder characterized by widespread musculoskeletal pain accompanied by fatigue, sleep disturbance, cognitive symptoms, and a variety of somatic complaints [1]. It is currently considered a prototypical nociplastic pain condition characterized by altered central pain processing, enhanced sensory amplification, and dysregulation of pain modulation pathways [2]. FM affects approximately 2–4% of the general population and occurs predominantly in women, with female-to-male ratios reported to range from 2:1 to 4:1 [2,3]. Beyond musculoskeletal manifestations, FM is increasingly recognized as a multisystem condition associated with functional disorders affecting different organ systems, particularly the gastrointestinal and genitourinary tracts [3]. Among these, irritable bowel syndrome (IBS) is one of the most frequently reported comorbidities in patients with FM, with studies reporting IBS in approximately 32–77% of individuals with FM [4].

IBS is a disorder of gut–brain interaction characterized by abdominal pain, altered bowel habits, visceral hypersensitivity, and dysregulation of bidirectional signaling between the enteric and central nervous systems [5]. Previous studies have suggested that IBS in FM is associated with greater symptom burden, poorer quality of life, psychological distress, and shared mechanisms of central sensitization [6,7]. The frequent coexistence of IBS and FM has led to the hypothesis that these conditions may represent overlapping manifestations of a shared centralized symptom spectrum rather than independent organ-specific disorders [7].

Lower urinary tract symptoms, including urgency, frequency, and features consistent with overactive bladder (OAB), are also commonly described in FM [8]. Previous studies have reported a high prevalence of lower urinary tract symptoms among women with FM, with approximately two-thirds of patients reporting at least one lower urinary tract symptom [9]. Similar to IBS, OAB is increasingly considered a disorder of altered visceral sensory processing rather than purely organ-specific dysfunction [10]. Both IBS and OAB share key pathophysiological mechanisms, including visceral hypersensitivity, central sensitization, dysregulation of afferent signaling, and altered brain–organ axis interactions [10,11,12]. Experimental and clinical studies have additionally demonstrated the presence of cross-organ sensitization between the bowel and bladder through convergent neural pathways, suggesting that dysfunction in one visceral organ may amplify symptom perception in another [11,12]. These shared mechanisms suggest that gastrointestinal and urinary symptoms in FM may not occur independently but rather reflect a broader pattern of visceral involvement. IBS and OAB were therefore specifically selected for concurrent evaluation because they represent common gastrointestinal and urinary manifestations associated with FM and share plausible mechanisms of visceral hypersensitivity, central sensitization, and bowel–bladder cross-organ sensitization.

Although IBS and lower urinary tract symptoms have each been described in patients with FM, data specifically evaluating the coexistence of bowel and bladder symptoms within the same FM cohort remain limited. In particular, whether concomitant IBS and OAB symptoms are associated with greater FM symptom burden has not been sufficiently clarified. Evaluating this overlap may help characterize multisystem symptom clustering in FM and may support a more integrated clinical assessment of gastrointestinal and urinary complaints.

Therefore, the aim of the present study was to evaluate the overlap between IBS and OAB in women with FM and to examine whether their coexistence is associated with differences in FM symptom severity.

## 2. Materials and Methods

### 2.1. Study Design and Data Collection

This cross-sectional observational study was conducted among women with FM evaluated at the Physical Medicine and Rehabilitation outpatient clinic between August and December 2025. Consecutive patients aged 18–65 years who fulfilled the 2016 American College of Rheumatology (ACR) diagnostic criteria for FM were included [13].

Patients with active urinary tract infection, known neurological disorders affecting bladder function, prior pelvic malignancy, or incomplete clinical data were excluded. Patients with alarm gastrointestinal symptoms suggestive of organic gastrointestinal disease, including gastrointestinal bleeding, unexplained weight loss, or previously diagnosed inflammatory gastrointestinal disorders, were also excluded when identified during clinical evaluation. All inclusion and exclusion criteria were predefined before initiation of the study and applied consistently throughout patient recruitment.

Demographic characteristics, including age, education level, and year of FM diagnosis, were recorded. Body mass index (BMI) values were recorded for all participants and were included in multivariable analyses to reduce the potential impact of body composition-related confounding. FM symptom burden was assessed using the Widespread Pain Index (WPI), Symptom Severity Scale (SSS), and Fibromyalgia Severity Score (GSS). The WPI evaluates the number of painful body regions, whereas the SSS assesses fatigue, sleep disturbance, cognitive symptoms, and somatic symptom severity. The GSS was calculated as the sum of WPI and SSS scores and was used as a composite indicator of overall FM severity. These instruments are widely used and validated measures for the assessment of FM symptom burden [13].

The presence of IBS was determined based on documented prior physician diagnosis or patient-reported previous physician diagnosis consistent with irritable bowel syndrome. Because the study was designed to evaluate previously recognized IBS as a clinical comorbidity rather than to establish a new diagnosis of IBS, prospective Rome IV screening was not incorporated into the original study protocol.

Lower urinary tract symptoms were evaluated using the Overactive Bladder Symptom Score (OABSS). The OABSS is a validated symptom-based questionnaire assessing urinary urgency, daytime frequency, nocturia, and urgency urinary incontinence, with higher scores indicating greater symptom severity [14]. The OABSS was analyzed as a continuous variable, and the presence of overactive bladder symptoms was defined as an OABSS score ≥ 3. Dysuria was recorded as a dichotomous variable (present/absent).

Patients receiving pharmacological treatments directed at lower urinary tract symptoms (e.g., antimuscarinics, β3-adrenoreceptor agonists) or IBS-directed therapies were not excluded, as these treatments were not specifically directed at FM symptom severity, which constituted the primary outcome of the present analysis.

Patients were categorized according to the presence of IBS and OAB symptoms. Subgroup analyses were performed based on the presence of IBS, OAB, and their coexistence. The study was approved by the Ümraniye Training and Research Hospital Scientific Research Ethics Committee (approval number: E-54132726-000-276063488, 9 May 2025) and conducted in accordance with the Declaration of Helsinki.

### 2.2. Statistical Analysis

Statistical analyses were performed using IBM SPSS Statistics for Windows, version 22.0 (IBM Corp., Armonk, NY, USA). Continuous variables were expressed as mean ± standard deviation (SD) or median (interquartile range [IQR]) according to data distribution, and categorical variables as number and percentage. Normality was assessed using the Shapiro–Wilk test. Homogeneity of variance was evaluated using Levene’s test.

Patients were categorized into four phenotypic groups according to the presence of IBS and OAB: neither condition, OAB only, IBS only, and concomitant IBS + OAB. Because only two patients had IBS without OAB, the IBS-only group was excluded from subgroup severity analyses due to the small cell size. Accordingly, subgroup comparisons were performed among the three remaining groups (neither condition, OAB only, and concomitant IBS + OAB; *n* = 72).

Comparisons of FM severity scores (WPI, SSS, and GSS) across groups were performed using one-way ANOVA for normally distributed variables or the Kruskal–Wallis test for non-normally distributed variables. When overall group differences were statistically significant, post hoc pairwise comparisons were performed using the Dunn–Bonferroni correction, and adjusted *p*-values were reported for all pairwise comparisons.

Categorical variables were compared using the Pearson chi-square test or Fisher’s exact test. The association between OAB and IBS was further evaluated by calculating odds ratios (ORs) with 95% confidence intervals (CIs).

To further evaluate the association between visceral symptom clustering and FM severity, multivariable linear regression analysis was performed using GSS as the dependent variable. A categorical visceral symptom clustering variable (neither condition [reference category], OAB only, and concomitant IBS + OAB) was included in the model together with age as a covariate. Unstandardized regression coefficients (B), 95% CIs, t-statistics, and *p*-values were reported. Overall model performance was reported using R^2^, adjusted R^2^, and the F-statistic with its corresponding *p*-value.

A post hoc power analysis was performed using G*Power (version 3.1.9.7) based on the observed differences in GSS scores between groups using the subgroup sample size (*n* = 72) and the observed effect size (η^2^, converted to Cohen’s f). Achieved statistical power (1 − β) was calculated at an alpha level of 0.05.

All tests were two-tailed, and a *p*-value < 0.05 was considered statistically significant.

## 3. Results

A total of 74 women with FM were included in the analysis. The mean age of the cohort was 44.9 ± 9.1 years, and the mean BMI was 22.8 ± 1.9 kg/m^2^. IBS was identified in 13 patients (17.6%), while OAB symptoms were present in 46 patients (62.2%). The demographic and clinical characteristics of the study population are summarized in Table 1.

A high proportion of IBS-positive patients also had OAB symptoms (84.6% vs. 57.4%). IBS prevalence was higher among OAB-positive than OAB-negative patients (23.9% vs. 7.1%), evaluated using Fisher’s exact test (OR = 4.56, 95% CI 0.93–22.24, *p* = 0.063). Although this association did not reach conventional statistical significance and the confidence interval crosses one, the magnitude of the odds ratio suggests a clinically plausible trend. The cross-tabulation of IBS and OAB overlap across the full cohort (N = 74) is detailed in Table 2.

To compare FM symptom severity across visceral symptom phenotypes, patients were categorized into three primary subgroups: IBS + OAB (*n* = 11), OAB only (*n* = 35), and neither condition (*n* = 26). Two patients presenting with IBS alone were excluded from subgroup comparisons due to the small cell count (*n* = 2) in this category, leaving a total of 72 patients for subgroup severity analyses.

Evaluation of FM severity indices demonstrated significant overall group differences for SSS (Kruskal–Wallis H = 29.921, *p* < 0.001) and GSS (Kruskal–Wallis H = 13.388, *p* = 0.001), whereas WPI scores did not differ significantly across groups (H = 2.954, *p* = 0.228).

Post hoc pairwise comparisons with Dunn–Bonferroni adjustment demonstrated that patients in the IBS + OAB subgroup had significantly higher GSS scores compared with both the Neither group (adjusted *p* = 0.001) and the OAB-only group (adjusted *p* = 0.003). Similarly, SSS scores were significantly higher in the IBS + OAB subgroup than in both the Neither group (adjusted *p* < 0.001) and the OAB-only group (adjusted *p* < 0.001). No significant differences in any severity index were observed between the OAB-only and Neither groups (adjusted *p* > 0.05 for all). Median scores and pairwise comparison statistics are summarized in Table 3.

In multivariable linear regression analysis adjusting for age, visceral symptom clustering was independently associated with higher FM severity (GSS). The overall regression model was statistically significant (F(3, 68) = 5.054, *p* = 0.003), accounting for 18.2% of the variance in FM severity (R^2^ = 0.182, Adjusted R^2^ = 0.146). The presence of concomitant IBS and OAB was a significant independent predictor of increased GSS (unstandardized B = 3.77, 95% CI 1.59–5.95, t = 3.44, *p* = 0.001), whereas OAB alone showed no independent association (unstandardized B = 0.58, 95% CI −1.09–2.25, t = 0.70, *p* = 0.490). Full model parameters are presented in Table 4.

Post hoc power calculations based on the subgroup sample size (N = 72) yielded an effect size of η^2^ = 0.165 (Cohen’s f = 0.444) for GSS comparisons, corresponding to an achieved statistical power (1 − β) of 92.0% at an alpha level of 0.05. For SSS comparisons, the achieved statistical power was 100.0% (η^2^ = 0.409, Cohen’s f = 0.832).

## 4. Discussion

We identified a substantial overlap between IBS and OAB symptoms in women with FM. A high proportion of patients with IBS also had OAB symptoms (84.6%), and IBS was more frequently observed among patients with OAB compared with those without (23.9% vs. 7.1%; OR = 4.56, 95% CI 0.93–22.24). However, this association did not reach statistical significance (*p* = 0.063) and should therefore be interpreted cautiously. Patients with concomitant IBS and OAB exhibited significantly higher SSS and GSS scores, whereas WPI did not differ significantly across visceral symptom groups. To our knowledge, studies specifically examining the coexistence of IBS and OAB and their association with FM symptom severity within the same cohort are limited. Together, these findings suggest that bowel–bladder comorbidity in FM may be associated with greater overall symptom burden, particularly in symptom severity and FM severity.

The co-occurrence of gastrointestinal and lower urinary tract symptoms is biologically plausible within the framework of central sensitization [15]. Both IBS and OAB are increasingly recognized as disorders involving altered visceral sensory processing, including heightened afferent signaling, impaired descending inhibitory control, and convergence within brain–gut–bladder neural pathways [15]. Given that FM is considered a prototypical centralized pain condition, the clustering of visceral symptoms may reflect a broader degree of central nervous system involvement rather than isolated organ-specific dysfunction [16].

Our findings add to previous literature by suggesting that the coexistence of IBS and OAB in FM may be associated with differences in overall symptom severity. Previous studies have consistently demonstrated a high prevalence of IBS among patients with FM, with reported rates ranging from approximately 32% to 80% [17]. The IBS prevalence observed in the present cohort (17.6%) was lower than in many previously published studies. Several factors may account for this discrepancy. First, IBS identification was based on prior physician diagnosis rather than active prospective screening using standardized Rome IV criteria, which may have resulted in under-ascertainment of IBS cases. Second, referral patterns at a Physical Medicine and Rehabilitation outpatient clinic may differ from those of gastroenterology-based or tertiary FM cohorts, where higher IBS prevalence has been reported. Together, these methodological differences may partially explain the lower prevalence of IBS observed in our cohort.

Lower urinary tract symptoms, including OAB, are also highly prevalent in FM, with reported rates of approximately 62% and a markedly higher frequency compared with non-FM populations [8,18]. However, these conditions have largely been evaluated separately. In our cohort, patients with combined bowel and bladder involvement exhibited significantly higher SSS and GSS scores, whereas WPI did not differ significantly across groups. Furthermore, the association between concomitant IBS and OAB and higher GSS remained significant after adjustment for age in multivariable analysis. These findings support the possibility that the coexistence of bowel and bladder symptoms may be associated with a broader pattern of symptom amplification in FM.

These findings may have implications for the clinical assessment of patients with FM. In routine practice, IBS and OAB are often managed as separate comorbid conditions, despite evidence from population-based studies indicating that these conditions frequently coexist [10]. Although the association between IBS and OAB itself did not reach statistical significance in our cohort, the presence of one condition may warrant consideration of symptoms of the other, particularly in patients with a high overall FM symptom burden. A more integrated assessment of gastrointestinal and urinary symptoms may therefore help identify patients with a higher overall symptom burden and support more comprehensive management strategies.

Recognition of bowel–bladder symptom clustering may also have potential therapeutic implications. Patients with concomitant IBS and OAB symptoms may represent a subgroup with greater multisystem symptom amplification and may potentially benefit from multimodal approaches targeting central pain processing in addition to organ-specific treatments. Such strategies may include pharmacological and behavioral interventions addressing central sensitization and cross-system symptom interactions. However, therapeutic interventions were not evaluated in the present study; therefore, any potential benefit of such multimodal approaches remains speculative and requires evaluation in prospective interventional studies.

The observed association between OAB and IBS (OR = 4.56, 95% CI 0.93–22.24; *p* = 0.063) should be interpreted as a non-significant finding rather than evidence of a confirmed association. The wide confidence interval, which included unity, also reflects the limited precision of this estimate, likely related to the small number of IBS-positive patients, particularly those without OAB. Accordingly, this finding requires confirmation in larger cohorts.

Several limitations should be considered. The cross-sectional design precludes conclusions regarding causality or temporal relationships between IBS and OAB. IBS status was based on prior diagnosis or patient report rather than standardized Rome IV criteria. The reliance on patient-reported or previously diagnosed IBS, without prospective application of standardized diagnostic criteria, is a meaningful limitation that may have resulted in both under- and misclassification of IBS cases. Application of Rome IV criteria in future studies would substantially improve diagnostic accuracy and comparability. OAB was defined based on symptom scores without urodynamic confirmation. The potential influence of medications affecting urinary or gastrointestinal symptoms (e.g., antimuscarinics, β3-adrenoreceptor agonists, antispasmodics) on OABSS scores was not evaluated, as medication use was not systematically recorded. In addition, the absence of a healthy control group limits the ability to determine whether bowel–bladder symptom clustering is specific to FM or reflects a broader phenomenon observed in other chronic symptom conditions. The sample size was modest, particularly for the IBS+OAB subgroup, and the very small IBS-only subgroup precluded its inclusion in subgroup severity analyses. Despite these limitations, the study also has notable strengths, including a well-characterized FM cohort assessed using standardized symptom indices and the simultaneous evaluation of gastrointestinal and urinary manifestations within the same population.

## 5. Conclusions

Irritable bowel syndrome is common in women with FM and shows a substantial overlap with OAB symptoms. FM patients with concomitant bowel and bladder involvement exhibit greater overall symptom burden, particularly higher SSS and GSS scores, compared with those without visceral symptoms, suggesting that bowel–bladder symptom clustering may be associated with a more symptomatic FM phenotype. The presence of either IBS or OAB may justify clinical evaluation for the other condition, as their coexistence may characterize patients with greater overall symptom burden and support consideration of a more integrated clinical approach. Further larger-scale prospective studies using standardized diagnostic criteria are needed to clarify the underlying mechanisms and clinical significance of these findings.

## Figures and Tables

**Table 1:** Demographic and clinical characteristics of the study population.

Variable	Value (*n* = 74)
Age, years	44.9 ± 9.1
BMI, kg/m^2^	22.8 ± 1.9
Education level, n (%)	
—Primary school	26 (35.1)
—Middle school	24 (32.4)
—High school	19 (25.7)
—University	5 (6.8)
Year of FM diagnosis	2019 (2015–2022)
WPI	14 (13–17)
SSS	7 (6–9)
GSS	16 (12–19)
IBS, n (%)	13 (17.6)
OAB, n (%)	46 (62.2)

Note: Values are presented as mean ± standard deviation or median (interquartile range), as appropriate. BMI: Body Mass Index; WPI: Widespread Pain Index; SSS: Symptom Severity Scale; GSS: Fibromyalgia Severity Score.

**Table 2:** Distribution of IBS according to OAB status.

	OAB (+) (*n* = 46)	OAB (−) (*n* = 28)
IBS (+)	11 (23.9%)	2 (7.1%)
IBS (−)	35 (76.1%)	26 (92.9%)

Note: Values are presented as n (%), with percentages calculated within OAB groups. Fisher’s exact test: OR = 4.56 (95% CI 0.93–22.24; *p* = 0.063). IBS: Irritable Bowel Syndrome; OAB: Overactive Bladder.

**Table 3:** Fibromyalgia severity across visceral symptom groups (N = 72).

Variable	IBS + OAB (*n* = 11)	OAB Only (*n* = 35)	Neither (*n* = 26)	*p*-Value *	I vs. N	I vs. O	O vs. N
WPI	14 (13.5–16)	14 (13–16)	13 (10–17.2)	0.228	0.365	1.000	0.561
SSS	9 (9–9)	6 (6–7)	7 (6–8)	<0.001	<0.001	<0.001	0.969
GSS	23 (22.5–25)	20 (19.5–22.5)	20.5 (16.5–22.5)	0.001	0.001	0.003	1.000

Note: Values are presented as median (interquartile range). * Overall *p*-values were obtained using the Kruskal–Wallis test. Pairwise comparisons were conducted using Dunn–Bonferroni post hoc correction. Pairwise comparison abbreviations are as follows: I vs. N, IBS + OAB versus Neither condition; I vs. O, IBS + OAB versus OAB only; O vs. N, OAB only versus Neither condition. *p*-values < 0.05 were considered statistically significant.

**Table 4:** Multivariable linear regression model for predictors of fibromyalgia severity (GSS).

Predictor	B	95% CI	t	*p*-Value
Intercept	17.63	14.10–21.17	9.95	<0.001
IBS + OAB (vs. Neither)	3.77	1.59–5.95	3.44	0.001
OAB only (vs. Neither)	0.58	−1.09–2.25	0.70	0.490
Age (years)	0.05	−0.03–0.13	1.21	0.231

Note: Dependent variable: Fibromyalgia Severity Score (GSS = WPI + SSS). The reference category for the visceral symptom group was the Neither condition group. Regression coefficients are presented as unstandardized coefficients (B). Overall model fit: R^2^ = 0.182, adjusted R^2^ = 0.146, F(3,68) = 5.054, *p* = 0.003.

## Data Availability

The data supporting the findings of this study are available upon reasonable request from the corresponding author. Access to qualitative data is restricted to protect participant confidentiality.

## Abbreviations

The following abbreviations are used in this manuscript:

BMI	Body mass index
FM	Fibromyalgia
IBS	Irritable bowel syndrome
OAB	Overactive bladder
OABSS	Overactive Bladder Symptom Score
WPI	Widespread Pain Index
SSS	Symptom Severity Scale
GSS	Fibromyalgia Severity Score
